# Synthesis and characterization of chitosan-functionalized nanostructured lipid carriers with temozolomide: cytotoxic effects and chromosomal instability in human glioblastoma cells

**DOI:** 10.1007/s00210-026-05365-y

**Published:** 2026-04-27

**Authors:** Helber Alves Negreiros, João Pedro Alves Damaceno do Lago, Victor Alves de Oliveira, Lia Raquel Alves Silva, Ana Carolina Lima de Melo, Igor Gabriel Barbosa de Sousa, Jefferson da Cruz Esteves, Sérgio Eduardo Matos Cazarotti Francisco, Samuel Lemos Paiva, João Pedro Crispim Guerra Rodrigues, Lucas Medeiros Martins Carvalho, Pedro Henricke Oliveira de Souza, Ana Flávia Chaves Uchôa, Anny Leticia Marinho Ramos Cardoso, Waleska Fernanda de Alencar Sales, Maria Hérika da Silva Bastos, Lidiane de Lima Feitoza, Dalton Dittz Júnior, Felipe Cavalcanti Carneiro da Silva, Francisco Humberto Xavier-Júnior, Paulo Michel Pinheiro Ferreira, João Marcelo de Castro e Sousa

**Affiliations:** 1 Postgraduate Program in Pharmaceutical Sciences, Toxicological Genetics Research Laboratory (Lapgenic), Federal University of Piauí, Teresina, Brazil; 2 Pharmaceutical Biotechnology Laboratory (BioTecFarm), Department of Pharmaceutical Sciences, Federal University of Paraíba, João Pessoa, Brazil; 3 Department of Biology, Federal University of Piauí, Teresina, Brazil; 4 Postgraduate Program in Pharmaceutical Sciences, Laboratory of Antineoplastic Pharmacology (LAFAN), Federal University of Piauí, Teresina, Brazil; 5 Laboratory of Experimental Cancerology (LabCancer), Department of Biophysics and Physiology, Federal University of Piauí, Teresina, Brazil

**Keywords:** Multiform glioblastoma, Chemoresistance, Nanostructured lipid carrier, Chitosan, Genotoxicity

## Abstract

Multiform glioblastoma (GBM) is the most aggressive primary brain tumor, associated with high heterogeneity, treatment resistance, and poor survival. Temozolomide (TMZ), although the main chemotherapeutic agent used, shows limited efficacy due to low solubility, chemical instability, and acquired resistance. In this context, nanostructured systems can enhance their antitumor efficacy. This study aimed to develop and characterize chitosan-functionalized nanostructured lipid carriers loaded with Temozolomide (NLCTQ), as well as to assess their biological activity in human glioblastoma cells (U87-MG). Lipid nanoparticles functionalized with chitosan were prepared by hot emulsification and sonication. Physicochemical characterization included DLS, zeta potential, FTIR, and HPLC for drug quantification and encapsulation efficiency. Biological activity was evaluated in U87-MG cells using the cell viability assay MTT and trypan blue, the comet assay, the spheroid model, fluorescence cell death, and the CBMN assay. The formulation presented a homogeneous nanometric size and positive zeta potential, although with moderate encapsulation efficiency (39%). Biological assays demonstrated that NLCTQ significantly reduced cell viability, overcoming the U87-MG cell line’s resistance to TMZ by achieving cytotoxicity at doses up to 20 times lower than free TMZ. Additionally, NLCTQ promoted the formation of biomarkers of chromosomal instability, such as micronuclei, bridges, and nuclear buds, which may explain the observed cytotoxic effects. Together, the results indicate that TMZ nanoencapsulation in NLCTQ enhances its antitumor efficacy, representing a promising strategy to overcome the limitations of conventional chemotherapy in glioblastoma treatment.

**Supplementary Information:**

The online version contains supplementary material available at 10.1007/s00210-026-05365-y.

## Introduction

Glioblastoma multiforme (GBM) is the most aggressive and malignant primary brain tumor. It is derived from astrocytes, star-shaped glial cells that support neurons in the central nervous system (CNS). Molecular classification of GBM considers mutations in isocitrate dehydrogenase (IDH) enzymes. IDH wild-type glioblastomas represent the majority of cases and are associated with higher aggressiveness and poorer prognosis, whereas IDH-mutant tumors, more frequently observed in younger patients, are linked to improved survival. As a grade IV astrocytoma, GBM is characterized by rapid proliferation, diffuse infiltration, and pronounced genetic heterogeneity. This heterogeneity contributes to resistance to conventional therapies, including surgery, radiotherapy, and chemotherapy, leading to an unfavorable prognosis. In newly diagnosed patients, median survival ranges from 12 to 15 months, and fewer than 30% of patients survive beyond two years (Obrador et al. 2024; Sipos et al. 2025).

Current standard treatment is associated with high relapse rates and limited therapeutic options for recurrent GBM, resulting in modest survival benefits. Since the introduction of the Stupp protocol in 2005, no substantial improvements in overall survival have been achieved (Sipos et al. 2025; Tan et al. 2020). Temozolomide (TMZ), when combined with standard therapy, has improved patient survival and remains the only chemotherapeutic agent that provides a clinically significant benefit in GBM treatment. However, intrinsic drawbacks, such as nonspecific toxicity, low solubility, and rapid hydrolysis under physiological conditions, limit its therapeutic efficacy. In addition, the presence of the blood–brain barrier and tumor-related factors, such as molecular heterogeneity and therapeutic resistance, further restrict its effectiveness (Iturrioz-Rodríguez et al. 2023). Therefore, strategies to enhance TMZ efficacy are essential to improve clinical outcomes.

In this context, the development of advanced drug delivery systems has emerged as a promising approach to overcome these limitations. Nanocarriers have demonstrated the ability to improve drug stability, enhance bioavailability, and promote sustained release, thereby increasing therapeutic efficiency while reducing systemic toxicity (Haider et al. 2020).

Among these systems, nanostructured lipid carriers (NLCs) represent an important advancement over solid lipid nanoparticles (SLNs). They consist of a mixture of solid and liquid lipids forming a partially crystallized matrix that enables higher drug loading capacity. NLCs also exhibit biocompatibility, low toxicity, scalability, and physicochemical stability, supporting their application in drug delivery, particularly in cancer therapy (Gomaa et al. 2022).

Compared to SLNs and polymeric nanoparticles (PNPs), NLCs loaded with TMZ have shown improved drug encapsulation, enhanced cellular delivery, and superior antitumor activity in both in vitro and in vivo models (Viegas et al. 2023; Qu et al. 2016). In addition, NLCs provide controlled drug release and improved brain delivery. Although PNPs can enhance TMZ stability and bioavailability, lipid-based systems generally exhibit more efficient drug transport and cellular interaction (Jatyan et al. 2022; Agrawal et al. 2020). Furthermore, NLCs can be functionalized with polymers, peptides, or antibodies to enable active targeting and further improve therapeutic performance (Oliveira et al. 2024; Aytekin et al. 2025).

In this context, chitosan (CS), a non-toxic, biodegradable, and biocompatible cationic polymer, has been widely used in drug delivery systems. CS exhibits unique properties, including mucoadhesiveness, controlled drug release capability, solubility in acidic aqueous media (allowing nanoparticle formation without toxic organic solvents), and the ability to modulate tight junctions. Surface modification of NLCs with CS confers additional advantages, such as the generation of a positive surface charge, enhanced cellular uptake via the proton-sponge effect, improved membrane interaction, and increased drug retention at the target site (Megahed et al. 2022; Gilani et al. 2021; Yu et al. 2019; Chirio et al. 2014).

Based on these considerations, the present study aimed to develop, characterize, and evaluate chitosan-functionalized nanostructured lipid carriers loaded with temozolomide (NLCTQ), with the goal of enhancing cytotoxic effects against glioblastoma cells and addressing key limitations of conventional chemotherapy.

## Materials and methods

### Preparation of nanoformulations

Nanostructured lipid carriers (NLCs) were produced using the hot emulsification method followed by sonication. Glyceryl distearate (Precirol® ATO 5) (2%, w/w) was used as the solid lipid, and medium-chain triglycerides (MCT) (4%, w/w) as the liquid lipid. Polysorbate 80 (Tween® 80) (3%, w/w) was used as the hydrophilic emulsifier, while soy lecithin (Lipoid® S100) (0.5%, w/w) was employed as the lipophilic emulsifier. The system was produced using Milli-Q® water as a solvent, in a final volume of 1 mL. For functionalization with CS, the solution was prepared by weighing 0.1% (w/v) CS, which was dispersed in 1 mL of water, followed by the addition of 2 µL of acetic acid solution. The mixture was kept under stirring, without heating, overnight until complete solubilization. Acidified CS at a concentration of 0.1% (w/v) was used in the aqueous phase. After weighing, the aqueous and oily phases were heated separately to 60 ± 5 °C for 10 min. Subsequently, the phases were combined and mechanically stirred using a magnetic stirrer at 900 rpm for 1 min. The resulting pre-emulsion was immediately sonicated at 70 W for 1 min to obtain empty chitosan-functionalized nanostructured lipid carriers (NLCQb). For drug-loaded formulations, temozolomide (TMZ) at concentrations of 1–3 mg/mL was solubilized in the oily phase, and chitosan-functionalized nanostructured lipid carriers loaded with TMZ (NLCTQ) were produced according to the same protocol previously described. All experiments were conducted in triplicate, and the results were expressed as mean ± standard deviation.

### Determination of particle size, polydispersity index, and Zeta potential

The mean hydrodynamic diameter and size distribution of the dispersions (expressed as the polydispersity index, PdI) were determined at 25 °C by dynamic light scattering (DLS) using a Zetasizer Lab instrument (Malvern Instruments). The scattering angle was fixed at 90°, and the samples were diluted 1:100 with Milli®-Q water immediately before the analysis.

The Zeta potential of the nanobiosystems was determined based on electrophoretic light scattering (ELS) measurements using the same instrument (Zetasizer Lab, Malvern Instruments). The dispersions were diluted (1:100) in 1 mmol/L NaCl solution before analysis. All experiments were conducted in triplicate, and the results were expressed as mean ± standard deviation.

### Fourier transform infrared spectroscopy (FTIR) characterization

Infrared analyses to identify functional groups were performed with individual components as well as with NLCs using a spectrophotometer (FTIR-ATR, Shimadzu® Cary 630). Data were collected in the spectral range of 4000 to 400/cm at 25 °C, with a resolution of 4/cm and 128 scans.

### Drug content determination and encapsulation efficiency

TMZ quantification was performed by HPLC–UV using an analytical calibration curve. Samples were analyzed using a chromatographic system (Alliance 2695, Waters, Milford, Massachusetts, USA) coupled to a 2998 photodiode array detector (DAD) (Waters, Milford, Massachusetts, USA) at a wavelength of 330 nm. A reverse-phase C18 column (250 × 4.6 mm, 5 μm, XBridge® Waters) protected by a column with the same stationary phase (20 mm × 4.6 mm) was used. Chromatographic separation was carried out in isocratic mode at 40 °C. The mobile phase consisted of a mixture of methanol and water acidified with 0.05% trifluoroacetic acid in an 80:20 (v/v) ratio. The flow rate was set at 1.2 mL/min, and the injection volume was 50 μL. Data acquisition and analysis were performed using Waters Empower® 3 software.

Three authentic analytical calibration curves were prepared for drug quantification at concentrations of 1, 2, 5, 10, 20, and 40 μg/mL in methanol. Diluted solutions were injected into the HPLC system, and peak areas were integrated. Linearity was evaluated by linear regression analysis using the least-squares method (Mulholland and Hibbert 1997).

The quantification of TMZ into the nanosystems (NLCs) was determined after the extraction of the nanoparticle core using an organic solvent. Briefly, 100 μL of the nanoparticle dispersion was added to a 10 mL volumetric flask containing methanol and vortexed for 2 min. The dispersion was transferred to a screw-cap centrifuge tube and subjected to an ultrasonic bath at 50 °C for 30 min. Subsequently, the tube was centrifuged at 10,000 rpm (~ 12,298 g) for 10 min. The supernatant was collected and filtered through a 0.22 μm membrane filter. The resulting solution was injected into the HPLC system under the chromatographic conditions described above. Peak areas were integrated, and the concentration of TMZ encapsulated in the nanosystems was calculated using previously obtained calibration curve equations.

Encapsulation efficiency (EE%) was determined by quantifying the free (non-encapsulated) TMZ in the nanosystems using ultrafiltration. Briefly, 500 μL of each TMZ-containing dispersion was added to the upper chamber of an ultrafiltration device (Amicon Ultra, Millipore, MWCO 10 kDa) and centrifuged in a microcentrifuge (Tomy, TX-160, Jakarta, Indonesia) at 5,000 rpm (~ 4,192 g) for 30 min. After centrifugation, 50 μL of the filtrate was diluted in acetonitrile and analyzed by HPLC. The amount of non-encapsulated drug was calculated by integrating the chromatographic peak areas using the calibration curve equation. After determining the drug concentrations, EE% was calculated using the equation, where total TMZ corresponds to the drug concentration obtained in the drug content assay and free TMZ corresponds to the TMZ concentration quantified in the filtrate.

$$EE\%=\frac{{TMZ}_{Total}-{TMZ}_{Free}}{{TMZ}_{Total}}x100$$

### Stability Analysis

The formulations (NLCTQ and NLCQb) were subjected to long-term stability studies under controlled temperature conditions. The NLCs were kept in sealed vials and evaluated over a period of 60 days at 4, 25, and 37 ºC. Throughout this interval, particle size, polydispersity index, and Zeta potential were measured according to the methods previously described.

### Cell culture

Human glioblastoma multiforme U87-MG cells were obtained from the Federal University of Minas Gerais (UFMG). The cell line was cultured in Dulbecco’s Modified Eagle’s Medium (DMEM) supplemented with 10% fetal bovine serum, penicillin 100 IU/mL, and streptomycin 100 μg/mL, and maintained at 37 °C in a humidified atmosphere containing 5% CO_2_ (Shel Lab CO_2_ Incubator, USA).

### Cytotoxic assays

The MTT assay {3-(4,5-dimethylthiazol-2-yl)−2,5-diphenyl tetrazolium bromide} was performed as described by Mosmann (1983), with minor modifications. Briefly, 5 × 10^3^ cells were seeded into 96-well plates and incubated with free TMZ (6.06 to 388 μg/mL) or TMZ-loaded nanostructured lipid carriers (NLCTQ, 1.56—100 μg/mL) or empty nanostructured lipid carriers (NLCQb) for 72 h at 37 °C in a 5% CO_2_ atmosphere. Subsequently, 20 μL of MTT solution (5 mg/mL) was added to each well and the plates were incubated for additional 4 h, followed by aspiration of the culture medium. Formazan crystals were dissolved in 100 μL of DMSO for 30 min, and the optical density was measured using a microplate spectrophotometer (GloMax Discover, Promega) at 550 nm.

### Trypan blue viability assay

Cell viability was also evaluated using the trypan blue exclusion test (Strober 2015). Human glioblastoma U87-MG cells were seeded into 24-well plates at a density of 2 × 10^4^ cells per well. After 24 h, the culture medium was replaced, and the cells were treated with free TMZ (5, 10, 50, 100, and 200 µg/mL), NLCTQ (2.5, 5, and 10 µg/mL), and NLCQb (2.5, 5, and 10 µg/mL). After 72 h of treatment, both treated and untreated cells were subjected to the assay. Non-viable cells were identified by blue staining and counted as dead cells. Cell counting was performed using a Neubauer chamber by optical microscopy at 400X magnification (Ferreira et al. 2022). The percentage of viable cells was calculated using the following formula:

$$Viable cells (\%) = \frac{total number of viable cells per aliquot}{total number of cells (viable and non-viable)}x100$$

### Cytotoxicity study of nanostructured lipid carriers (NLCs) in a three-dimensional spheroid cell culture model

Briefly, 25 μL droplets of a U87-MG cell suspension (6 × 10^4^ cells/mL) were placed on the lid of a sterile disposable Petri dish. The lid was then inverted over the Petri dish, allowing cell accumulation at the liquid–air interface, followed by incubation for 72 h to promote spheroid formation (Duval et al. 2017). The hanging drop method formed spheroids which transferred to a 48-well plate, one spheroid per well, and the plate was incubated for 60 min at 37 °C in a 5% CO_2_ atmosphere to ensure spheroid adhesion. After treatment with free TMZ and TMZ-loaded NLCs for 48 h, spheroids were registered using an inverted microscope equipped with ZEN 2 Pro software (version 2.0) at 0 h and 48 h to evaluate spheroid size and cell migration radius (Proença et al. 2019) by ImageJ software.

### Alkaline comet assay

Primary DNA damage, including single- and double-strand breaks and alkali-labile sites, induced by TMZ and formulations on U87-MG glioblastoma cells was evaluated using the standard alkaline comet assay (Møller et al. 2020). Following the treatments, cells were centrifuged for 10 min at 1200 × g. The supernatant was discarded, and 20 µL of the remaining cell suspension was mixed with 80 µL of freshly prepared 0.9% low-melting-point (LMP) agarose (agarose concentration of 0.72%). Forty microliters of this suspension were placed onto microscope slides previously coated with a layer of 1% normal-melting-point agarose and covered with 20 × 20 mm coverslips. Agarose solidification was allowed for 15 min on ice. Subsequently, the coverslips were removed, and the slides were immersed in lysis solution (NaOH 250 mM, Tris–HCl, Na₂ EDTA 100 mM, NaCl 2.5 M, pH 10, and Triton X-100 1%) at 4 °C overnight in the dark to prevent additional DNA damage (Valdiglesias et al. 2021).

After lysis, the slides were placed in a horizontal electrophoresis chamber on ice and incubated for 20 min in the dark in freshly prepared alkaline electrophoresis solution (NaOH 300 mM, Na₂EDTA 1 mM, pH > 13) to allow DNA unwinding. Electrophoresis was performed for 20 min at 0.83 V/cm. Thereafter, the slides were washed 3 times (5 min each) with a neutralization solution (Tris–HCl 0.4 M, pH 7.5) and dried at room temperature in the dark. The preparations were stained with silver staining solution (sodium carbonate, 5 g; distilled water, 100 mL; ammonium nitrate, 0.1 g; silver nitrate, 0.1 g; tungstosilicic acid, 0.25 g; and formaldehyde, 150 µL). The slides were stored in a sealed, humidified box at 4 °C to prevent agarose gel dehydration.

Slides were photomicrographically analyzed at 400X magnification under an optical microscope, and the results were expressed as damage index (DI) and damage frequency (DF) (100 cells analyzed in triplicate per treatment). The DI was calculated using the formula: DI = Σ (number of cells in each damage class × damage class), ranging from 0 to 400, and DF was calculated using the following formula: DF = 100 − number of class 0 cells.

### Cytokinesis-block micronucleus assay

To evaluate mutagenicity and cell death of U87-MG cells, the cytokinesis-block micronucleus (CBMN) assay was performed according to Fenech (2007), with minor adaptations. Tumor cells were incubated for 44 h at 37 ± 1 °C. After this period, cytochalasin B (12 μg/mL; Sigma, St. Louis, MO, USA) was added to the cultures treated with TMZ (50 and 100 µg/mL), NLCQb (2.5 and 5 µg/mL) or NLCTQ (2.5 and 5 µg/mL). After a total of 72 h, cultures were transferred to tubes, centrifuged at 1200 rpm for 10 min, and cell pellets were suspended in 1.5 mL of sodium citrate solution 1%, followed by another centrifugation. Subsequently, 5 mL of fixative solution (methanol: acetic acid, 3:1) and one drop of formaldehyde were added to the tubes. This procedure was repeated twice using the 3:1 fixative without formaldehyde. Finally, the supernatant was discarded, and 2–4 drops of the cell suspension were placed onto microscope slides and stained with Giemsa 3% solution for 10 min. The slides were previously blindly analyzed using optical microscopy (1000X magnification). Cytogenetic damage (micronuclei, nuclear buds, and nucleoplasmic bridges) and death events (apoptosis and necrosis) were registered in 1000 cells per slide (*n =* 3).

The nuclear division index (NDI) and the nuclear division index with cytotoxicity (NDIC) were counted in U87-MG-dividing cells. Estimations for cytotoxicity assessment were performed using the following formulas:

$$\begin{array}{c}\text{NDI }=\frac{\mathrm{M}1 + 2\text{X }(\mathrm{M}2) + 3\text{X }(\mathrm{M}3) + 4\text{X }(\mathrm{M}4)}{\text{Total number of cells}}\\ \text{NDIC }=\frac{(\text{Apoptosis }+\text{ Necrosis }+\text{ M}1 + 2\text{x }(\mathrm{M}2) + 3\text{x }(\mathrm{M}3) + 4\text{x }(\mathrm{M}4)}{\text{Total number of cells}}\end{array}$$

where: “Apoptosis” represents the number of apoptotic cells; “Necrosis” represents the number of necrotic cells; “M1” represents the number of viable cells with one nucleus; “M2” represents the number of viable cells with two nuclei; “M3” represents the number of viable cells with three nuclei; “M4” represents the number of viable cells with four nuclei; and “Total number of cells” represents the total number of cells analyzed, corresponding to 1,000 cells per slide (*n =* 3).

### Analysis of cell death

To evaluate the type of cell death (apoptosis or necrosis), dual staining with acridine orange (AO) and propidium iodide (PI) was employed (Gao et al. 2020; Bankó et al. 2021). Cell identification criteria followed the evaluations described below: (a) viable cells exhibit a light green nucleus with intact cellular structure; (b) early apoptotic cells display a light green nucleus with evident chromatin condensation; (c) late apoptotic cells show dense orange areas (green/red) of chromatin condensation and membrane blebbing; and (d) necrotic cells present a red nucleus (Renvoizé et al. 1998; Tan and Norhaizan 2019).

U87-MG cells were seeded in 12-well plates and incubated for 72 h at a density of 2 × 10^4^ cells/mL. After treatments (TMZ: 50 and 100 µg/mL; NLCQb: 2.5 and 5 µg/mL; NLCTQ: 2.5 and 5 µg/mL), cells were collected by centrifugation (1200 rpm, 24 °C, 10 min), suspended in phosphate-buffered saline (PBS), and stained with 20 µL of AO/PI solution (10 µg/mL). Freshly stained cells were observed under a fluorescence microscope (Leica DM2500). The percentages of viable, early apoptotic, late apoptotic, and necrotic cells were determined (*n =* 200 cells per slide, *n =* 3).

### Statistical analysis

Statistical comparisons were performed by one-way or two-way analysis of variance (ANOVA), followed by Tukey or Bonferroni, as well as by Student *t*-test, considering *p* < 0.05 (GraphPad Prism version 9.5.1). Data are expressed as mean ± standard deviation (S.D.). All experiments were performed in duplicate, representing independent biological evaluations on alternate days or weeks, with each concentration tested in triplicate.

## Results

### Physicochemical features

**Fig. 1:**
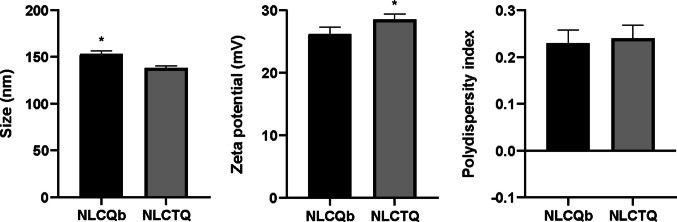
Hydrodynamic diameter (**A**), polydispersity index (**B**), and Zeta potential (**C**) of NLCQb and NLCTQ. Statistical analysis was performed using the Student *t*-test to compare differences between the experimental groups NLCQb (black chitosan-functionalized nanostructured lipid carriers) and NLCTQ (temozolomide-loaded chitosan-functionalized nanostructured lipid carriers). Data are expressed as mean ± S.D. from three independent experiments (*n = *3). * indicates *p <* 0.05 between the groups. The figure demonstrates the comparative effect between unloaded and drug-loaded nanosystems, highlighting the impact of temozolomide incorporation on the evaluated parameter

Both formulations presented hydrodynamic diameters below 155 nm, an optimal measure for enhanced penetration and delivery across the blood–brain barrier (Fig. 1). The NLCTQ formulation containing TMZ presented a mean diameter of 138.2 ± 1.4 nm, suggesting potential differences in biological interactions when compared to NLCQb (153.4 ± 2.2 nm). In addition, both formulations showed low polydispersity index values (0.23 ± 0.02 for NLCQb and 0.24 ± 0.02 for NLCTQ), confirming the homogeneity of the formulations.

NLCTQ exhibited a bimodal size distribution, with two distinct populations centered at approximately 31 nm and 185 nm, suggesting the coexistence of smaller nanoparticles and larger aggregates with structural heterogeneity. In contrast, NLCQb displayed a predominantly monomodal distribution centered around 200 nm, indicating a more uniform particle population. Despite this difference in distribution profiles, both formulations maintained moderate polydispersity (PdI ~ 0.23), reinforcing their overall physicochemical suitability. A more detailed analysis of size distribution profiles is provided in supplementary Figure S1 and Table S1.

Zeta potential measurements also revealed satisfactory values (> 20 mV) for both NLCQb (+ 26.2 ± 0.8 mV) and NLCTQ (+ 28.6 ± 0.6 mV) formulations, ensuring colloidal stability due to electrostatic repulsion between particles (Fig. 1). Complementary data presented in Supplementary Figure S2 and Table S2 further highlight the surface charge characteristics of the system. NLCTQ exhibited a positive Zeta potential of + 28.6 mV, consistent with good colloidal stability, while also showing a relatively high Zeta deviation (12.99 mV) and the presence of multiple Zeta potential populations. These findings suggest heterogeneity in surface charge distribution, a possibility arising from the interaction between formulation components or differential surface organization.

Overall, the combined analysis of particle size distribution and Zeta potential profiles indicates that, although both formulations exhibit suitable physicochemical properties for nanocarrier applications, NLCQb presents a more uniform size distribution, whereas NLCTQ demonstrates higher structural and surface heterogeneity, which may influence its biological performance.

### Infrared spectroscopy results

**Fig. 2:**
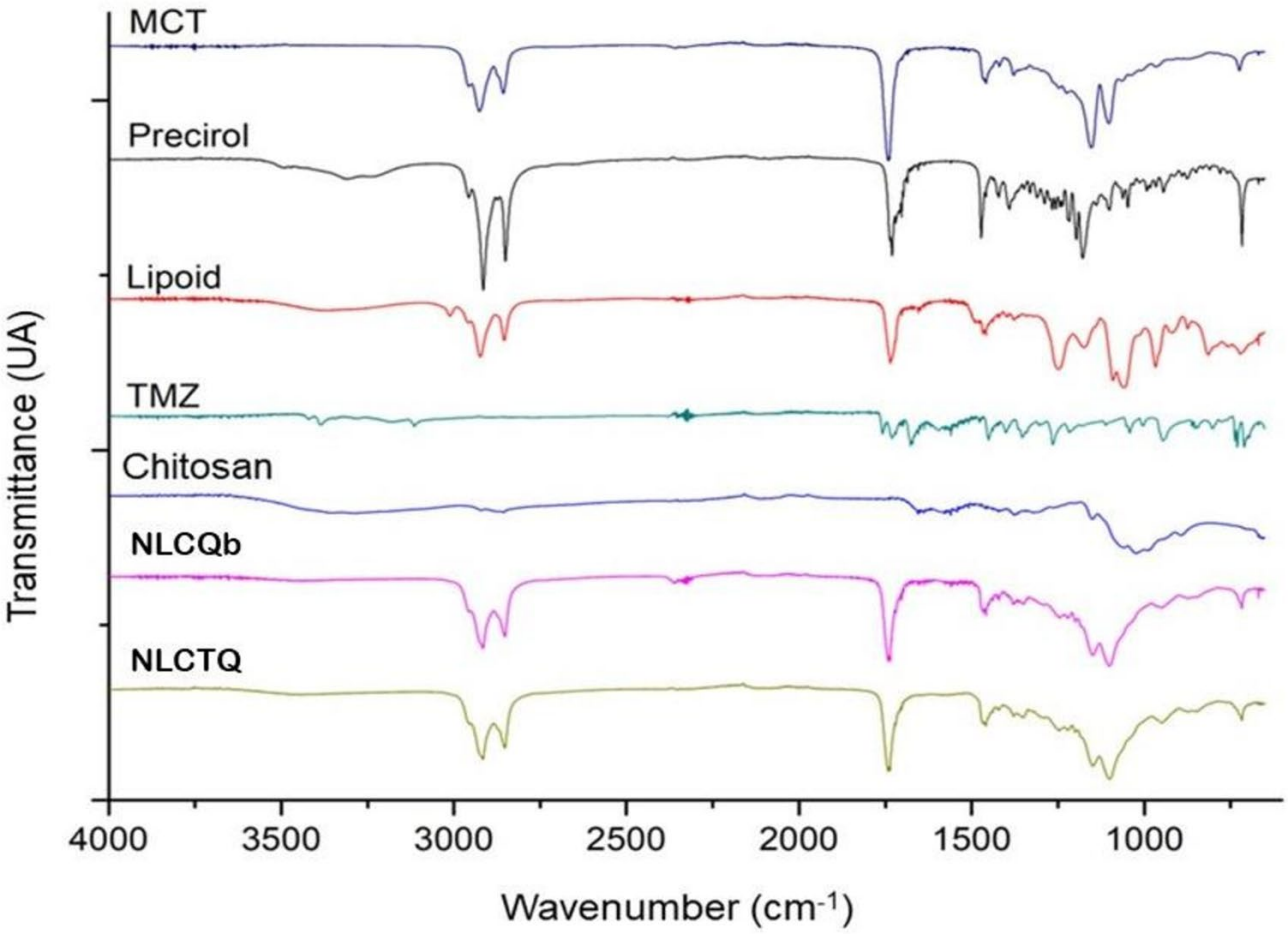
Infrared spectrum of NLCTQ, NLCQb, and the components used in their production. Fourier-transform infrared spectra of raw materials and nanostructured lipid carrier formulations, with curves identified according to their respective colors: medium-chain triglycerides (MCT, dark blue), glyceryl distearate (Precirol® ATO 5, black), soybean lecithin (Lipoid® S100, red), temozolomide (TMZ, cyan), chitosan (blue), blank chitosan-functionalized nanostructured lipid carriers (NLCQb, magenta), and temozolomide-loaded chitosan-functionalized nanostructured lipid carriers (NLCTQ, olive green)

FTIR spectroscopic analysis allowed the identification of characteristic bands of the individual componentes, as well as of the NLCQb and NLCTQ formulations (Fig. 2). Both formulations exhibited absorption bands at approximately 2920 cm⁻^1^ and 2850 cm⁻^1^, attributed to the asymmetric and symmetric C–H stretching vibrations of –CH₂ groups in alkyl chains, confirming the presence of the lipid matrix. The band observed at approximately 1735 cm⁻^1^ in both formulations corresponds to the C = O stretching vibration of ester groups present in Precirol and Lipoid® S100. In addition, both formulations showed a band at approximately 1240 cm⁻^1^, attributed to the P = O stretching vibration of the phosphate group of Lipoid® S100.

In lipid-based systems, FTIR analysis often shows overlapping bands among carbonyl, phosphate, and amide groups, which limits its ability to conclusively confirm drug encapsulation in the absence of significant band shifts. Therefore, the encapsulation was achieved through the determination of encapsulation efficiency by HPLC.

### Drug content and encapsulation efficiency

The quantification of TMZ in the NLCTQ formulation by HPLC revealed a concentration of 1.23 ± 0.10 mg/mL. In addition, the encapsulation efficiency was 39%.

### Stability Analysis

**Fig. 3:**
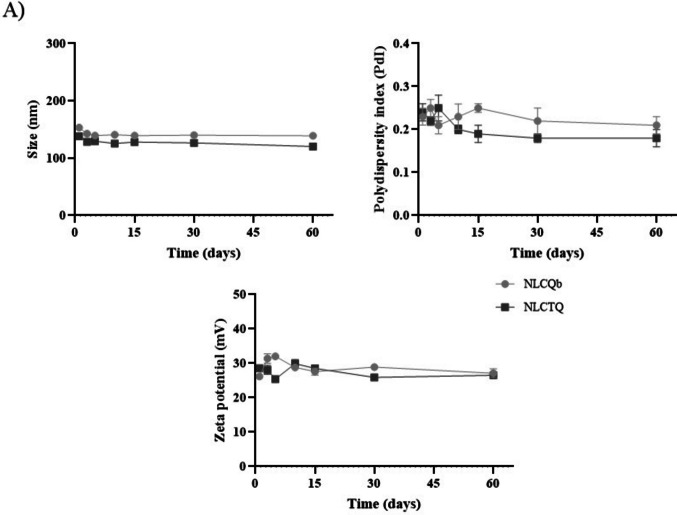

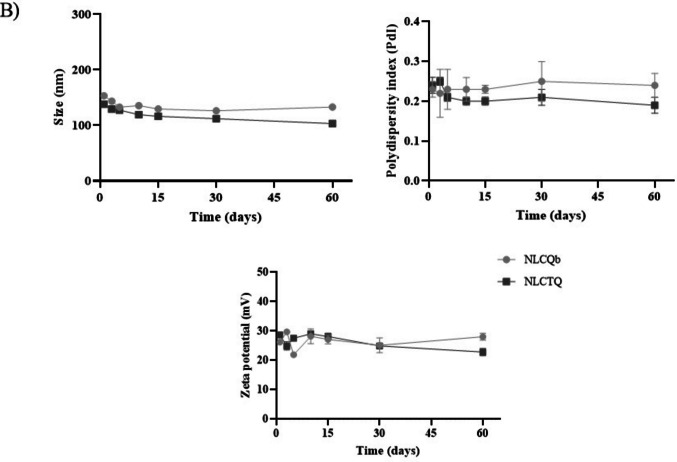

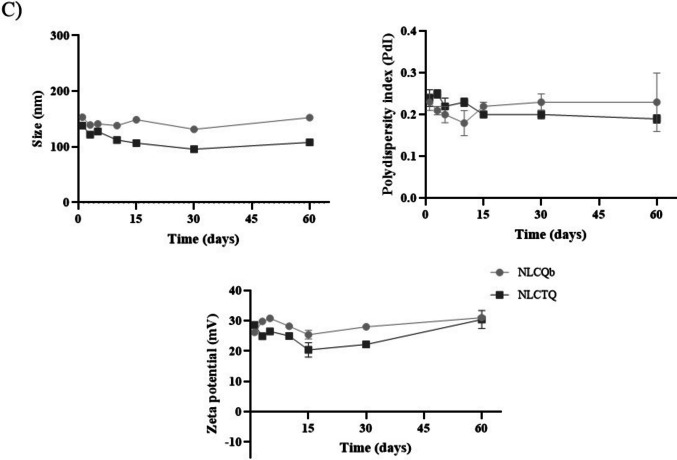
**A.** Physicochemical stability of NLCQb and NLCTQ formulations over 60 days at 4 °C, based on particle size, polydispersity index (PdI), and zeta potential (ZP). Data are expressed as mean ± standard deviation (SD) from three independent experiments (*n = *3). **B.** Physicochemical stability of NLCQb and NLCTQ formulations over 60 days at 25 °C, based on particle size, polydispersity index (PdI), and zeta potential (ZP). Data are expressed as mean ± standard deviation (SD) from three independent experiments (*n = *3). **C.** Physicochemical stability of NLCQb and NLCTQ formulations over 60 days at 37 °C, based on particle size, polydispersity index (PdI), and zeta potential (ZP). Data are expressed as mean ± standard deviation (SD) from three independent experiments (*n =* 3)

In this study, the chitosan-functionalized formulation (NLCTQ) and chitosan-functionalized without TMZ formulation (NLCQb) were evaluated in terms of size, polydispersity index (PdI), and Zeta potential over 60 days at refrigerated (4 ºC) (Fig. 3A), room temperature (25 ºC) (Fig. 3B), and incubator (37 ºC) (Fig. 3C).

Throughout the 60-day storage period at 4 ºC (Fig. 3A), the formulations exhibited distinct profiles regarding average particle size, PdI, and ZP. The NLCQb formulation showed initial particle size values of 153.4 ± 2.2 nm (day 1), followed by a reduction in the first few days and subsequent stabilization around 139.4 ± 1.5—142.7 ± 4.0 nm between days 5 and 60, indicating maintenance of system homogeneity over time. In turn, the NLCTQ formulation showed a gradual and continuous decrease in particle size during storage, starting from 138.2 ± 1.4 nm on day 1 and reaching 120.4 ± 0.6 nm at the end of 60 days. Regarding the polydispersity index (PdI), both formulations showed low values and a slight reduction over time, varying from 0.23 ± 0.02 to 0.21 ± 0.02 (NLCQb) and from 0.24 ± 0.02 to 0.18 ± 0.02 (NLCTQ). Regarding Zeta potential (ZP), NLCQb showed positive values ranging from 31.4 ± 1.4 to 28.9 ± 0.1, reaching 27.1 ± 1.3 at the end of the period, while NLCTQ varied from 28.6 ± 0.6 on day 1 to 26.5 ± 0.4 on day 60. The data presented show a consistent size distribution and absence of significant aggregation, remaining relatively stable over time, which suggests preservation of the colloidal stability of the dispersions during refrigerated storage.

During the 60-day storage period of 25ºC (Fig. 3B), the formulations exhibited distinct behaviors in terms of particle size, PdI, and ZP. The NLCQb formulation showed an initial reduction in particle size from 153.4 ± 2.2 nm to values around 126–133 nm at the end of the period, with slight fluctuations, indicating maintenance within the nanoscale range. In contrast, NLCTQ displayed a continuous decrease from 138.2 ± 1.4 nm to 103.4 ± 0.3 nm over 60 days, suggesting progressive structural reorganization. Regarding the polydispersity index (PdI), NLCQb remained relatively stable, varying from 0.23 ± 0.02 to 0.24 ± 0.03, while NLCTQ showed a reduction from 0.24 ± 0.02 to 0.19 ± 0.02, indicating improved uniformity. As for the Zeta potential (ZP), NLCQb remained stable, ranging from 26.2 ± 0.8 to 28.0 ± 1.2, whereas NLCTQ decreased from 28.4 ± 0.6 to 22.8 ± 1.2 over time. Overall, both formulations maintained nanoscale size and low PdI values without evidence of significant aggregation, although NLCTQ exhibited more pronounced physicochemical changes at 25ºC.

Under storage at 37ºC over a 60-day period (Fig. 3C), the formulations exhibited distinct behaviors in terms of particle size, PdI, and zeta potential. The NLCQb formulation maintained a nearly constant particle size, varying from 153.4 ± 2.2 nm (day 1) to 152.5 ± 2.5 nm (day 60), while NLCTQ showed a progressive reduction from 138.2 ± 1.4 nm to 108.1 ± 0.6 nm, with values dropping below 100 nm at day 30. The PdI remained stable for NLCQb (0.23 ± 0.02 to 0.23 ± 0.07), whereas NLCTQ exhibited a reduction from 0.24 ± 0.02 to 0.19 ± 0.01, indicating improved uniformity. Both formulations showed fluctuations in zeta potential over time, with NLCQb presenting values of 29.8 ± 0.7 mV (day 3), 28.0 ± 1.0 mV (day 30), and 31.0 ± 0.9 mV (day 60), and NLCTQ showing 24.9 ± 1.0 mV, 22.2 ± 0.8 mV, and 30.4 ± 3.0 mV at the same time points. Despite these variations, both formulations exhibited higher zeta potential values at day 60 compared to day 3. Overall, the increase in zeta potential values over time indicates enhanced electrostatic repulsion between particles, contributing to improved colloidal stability of the formulations.

### Cytotoxic action on glioblastoma cells

Curve concentration-cellular responses revealed an IC50 value of 266 µg/mL for free TMZ in U87-MG glioblastoma cells. When incorporated into chitosan-functionalized nanostructured lipid carriers, TMZ (NLCTQ) presented a significantly lower IC50 value compared to free TMZ. However, the empty chitosan-functionalized nanostructured lipid carriers (NLCQb) also showed significant cytotoxicity (Table 1).

**Table 1:** Cytotoxicity by the MTT method of free temozolomide (TMZ), empty nanostructured chitosan lipid carriers (NLCQb), and nanostructured chitosan lipid carriers loaded with temozolomide (CLTNQ) on the U87-MG glioblastoma cell line

Treatment	Inhibitory concentration (IC_50_)
NLCQb	11.65 µg/mL (9.50 to 14.15 µg/mL)
NLCTQ	4.79 µg/mL (3.298 to 7.249 µg/mL)
TMZ	266 µg/mL (247 to 301 µg/mL)
CS	1691 µg/mL (1498 to 1953 µg/mL)

Data are expressed as IC_50_ (95%, C.I.) from three independent experiments (*n =* 3).

### Cellular viability

Cell viability following NLCTQ treatment decreased significantly at concentrations of 5 µg/mL (34.92 ± 6.02%) and 10 µg/mL (0 ± 0%) when compared with the negative control (98.11 ± 0.63%) (Table 2), as seen for the NLCQb formulation at 5 µg/mL (81.33 ± 6.16%) and at 10 µg/mL (38.89 ± 7.83%) when compared with the negative control (Table 2). In contrast, only the highest concentration of TMZ (200 µg/mL: 66.35 ± 2.26%) reduced cellular viability. NLCQb and NLCTQ at 5 and 10 µg/mL exhibited significantly higher cytotoxicity when compared with free TMZ at similar concentrations (Table 2, *p < *0.05).

**Table 2:** Cell viability of U87-MG glioblastoma cells determined by the Trypan Blue assay

**Treatment**	**Concentration** (µg/mL)	**Cell viability** (%)
**Negative Control**	-	98.11 ± 0.63
Chitosan-functionalized nanostructured lipid carriers (NLCQb)	2.5	88.82 ± 3.6
	5	81.33 ± 6.16^ab^
	10	38.89 ± 7.83^ab^
Temozolomide-loaded chitosan-functionalized nanostructured lipid carriers (NLCTQ)	2.5	90.13 ± 0.99
	5	34.92 ± 6.02^ab^
	10	0 ± 0^ab^
Temozolomide	5	93.97 ± 1.46
	10	92.69 ± 0.60
	50	93.25 ± 1.68
	100	90.03 ± 0.87
	200	66.35 ± 2.26^a^

Data are expressed as mean ± S.D. from three independent experiments (*n =* 3). Statistical analysis was performed using one-way ANOVA followed by Tukey. ^a^ *p <* 0.05 when compared with the negative control; ^b^*p <* 0.05 when compared with free temozolomide at concentrations of 5 and 10 µg/mL.

### Cytotoxicity of free and nanoencapsulated TMZ upon three-dimensional spheroid U87-MG cells

**Fig. 4:**
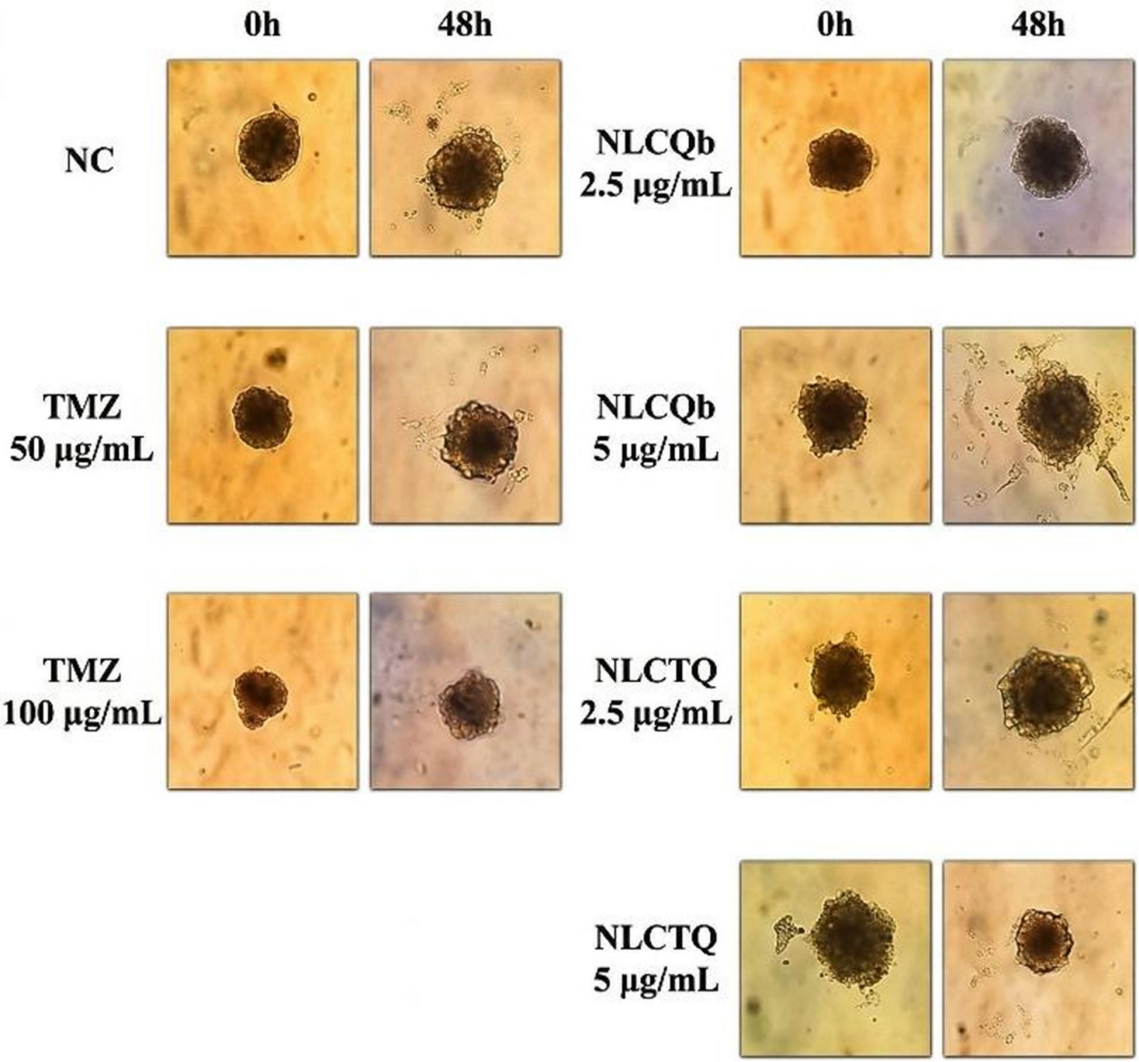
Representative images of human glioblastoma U87-MG cell spheroids incubated with TMZ, NLCQb, and NLCTQ for 48 h of treatment. DeeVid AI was used to improve spheroid clarity

The cytotoxic effects on spheroids induced by treatments with free TMZ, NLCQb, and NLCTQ are shown in Fig. 4.

**Fig. 5:**
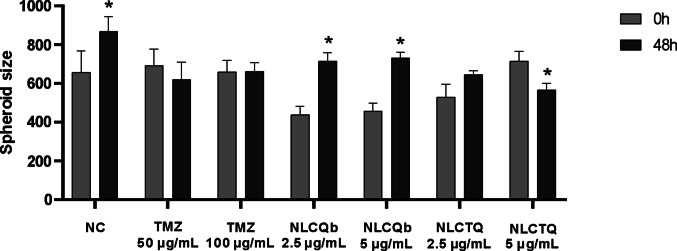
Cytotoxicity analysis of free and encapsulated temozolomide in nanostructured lipid carriers in a three-dimensional spheroid culture formed by human glioblastoma U87-MG cells after 48 h of treatment. Data are expressed as mean ± S.D. from three independent experiments (*n =* 3). Statistical analysis was performed using two-way ANOVA followed by Bonferroni. * Indicates a significant difference (*p <* 0.05) compared with the baseline time point (0 h) for each treatment

Figure 5 shows the evaluation of the spheroid sizes at 0 and 48 h after treatment with free TMZ (50 and 100 µg/mL), NLCQb (2.5 and 5 µg/mL), or NLCTQ (2.5 and 5 µg/mL). Only cells treated with NLCTQ 5 µg/mL exhibited a significant reduction (*p < *0.05) in spheroid size after 48 h [from 714.40 ± 51.12 µm (0 h) to 567.00 ± 33.56 µm (48 h)].

### TMZ and NLCTQ genotoxicity effects

**Fig. 6:**
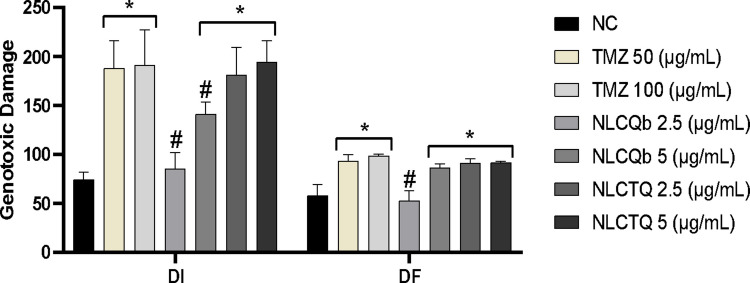
Damage index and damage frequency values in U87-MG glioblastoma cells treated with free temozolomide and temozolomide incorporated into nanostructured lipid carriers at different concentrations. Data are expressed as mean ± S.D. from three independent experiments (*n = *3). Statistical analysis was performed using one-way ANOVA followed by Tukey. * indicates a statistically significant difference (*p <* 0.05) compared with the negative control (NC); ^#^ indicates a statistically significant difference (*p < *0.05) between NLCQb and NLCTQ at the same concentration

All concentrations of TMZ and NLCTQ, as well as the 5 µg/mL concentration of NLCQb, induced genotoxicity in human U87-MG glioblastoma cells, as evidenced by increased damage index (DI) and damage frequency (DF) when compared with the negative control (NC). The NC group presented a DI of 74.33 ± 7.57 and a DF of 58.00 ± 11.14, whereas treatment with TMZ resulted in markedly higher values (TMZ 50 µg/mL: DI 188.00 ± 28.16, DF 93.00 ± 6.56; TMZ 100 µg/mL: DI 191.00 ± 36.50, DF 98.33 ± 2.08) (Fig. 6). Similarly, NLCTQ induced pronounced genotoxic effects at both concentrations evaluated (2.5 µg/mL: DI 181.00 ± 28.58, DF 91.00 ± 4.36; 5 µg/mL: DI 194.33 ± 21.50, DF 91.33 ± 1.53). In contrast, NLCQb at 2.5 µg/mL did not induce genotoxicity compared with the NC (DI 85.00 ± 16.82, DF 52.33 ± 10.69), while NLCQb at 5 µg/mL produced moderate genotoxicity (DI 140.67 ± 13.01, DF 86.33 ± 4.16). Furthermore, NLCTQ at both concentrations (2.5 and 5 µg/mL) exhibited significantly higher genotoxicity than NLCQb at the same concentrations (Fig. 6).

**Fig. 7:**
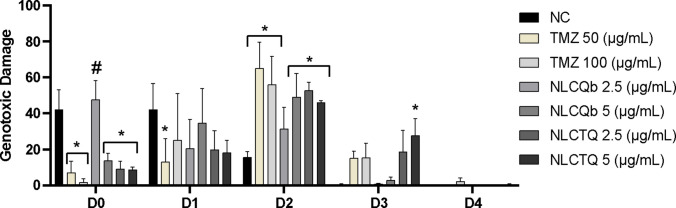
Quantitative variation of damage types (D0-D4) for the comet assay in the U87-MG cell line. Data are expressed as mean ± S.D. from three independent experiments (*n =* 3). Statistical analysis was performed using one-way ANOVA followed by Tukey. * indicates a significant difference (*p < *0.05) compared with the negative control (NC); ^#^ indicates a significant difference (*p < *0.05) between NLCQb and NLCTQ at the same concentration

In damage class D0, all treatments, except NLCQb 2.5 µg/mL (47.67 ± 10.69), presented a significantly reduced frequency of D0 cells compared to the negative control (42.00 ± 11.14). In D1, only TMZ 50 µg/mL (13.00 ± 13.08) showed a statistically significant damage reduction compared to the NC (42.00 ± 14.53). In D2, all treatments showed elevated DNA damage compared to the NC (15.67 ± 3.06), except the NLCQb at 2.5 µg/mL (31.33 ± 12.06). In D3, NLCTQ 5 µg/mL (27.67 ± 9.50) was the only treatment that showed an increase in DNA damage compared with the NC (0.33 ± 0.58). None of the treatments showed class D4 damage (Fig. 7).

### Mutagenicity and cytotoxicity on U87-MG cells

**Fig. 8:**
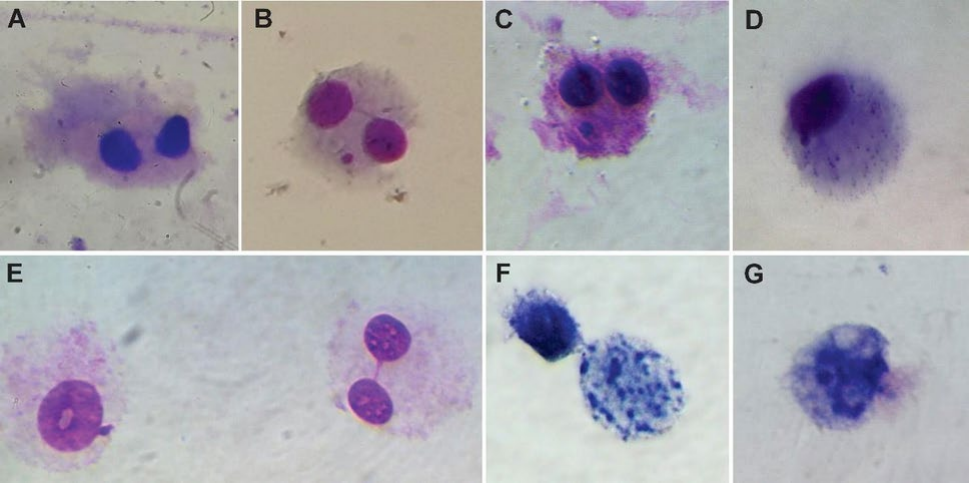
Morphological profile of human glioblastoma cells (U87-MG) treated with free TMZ, NLCQb, and NLCTQ and analyzed by the cytokinesis-blocked micronucleus assay. A) Untreated binucleated cell; B) Binucleated cell with nucleoplasmic bridge and micronucleus treated with TMZ 100 μg/mL; C) Binucleated cell with micronucleus treated with NLCTQ 2.5 μg/mL; D) Mononucleated cell with nuclear bud treated with TMZ 50 μg/mL; E) Mononucleated cell with nuclear bud (on the left) and a cell with nucleoplasmic bridge (on the right) after treatement with NLCTQ 5 μg/mL; F) Cell treated with NLCTQ 2.5 μg/mL under apoptosis (arrow); G) Cell treated with NLCTQ 2.5 μg/mL under necrosis

**Fig. 9:**
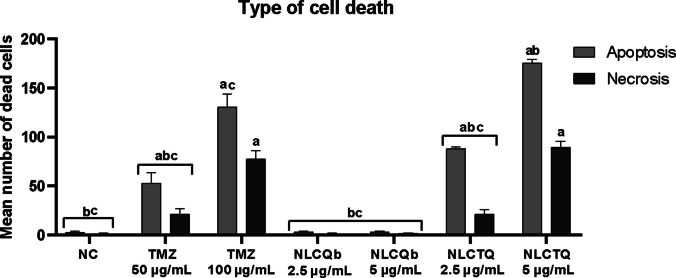
Results of cytokinesis-blocked micronucleus assays. Data are expressed as mean ± S.D. from three independent experiments (n = 3). NC: untreated cells. TMZ: temozolomide 50 and 100 μg/mL. NLCQb: empty chitosan-functionalized nanostructured lipid carriers (2.5 and 5 μg/mL). NLCTQ: chitosan-functionalized nanostructured lipid carriers loaded with temozolomide (2.5 and 5 μg/mL).^a^ *p <* 0.05 compared with the negative control by one-way ANOVA followed by Tukey; ^b^ *p < *0.05 compared with TMZ (100 μg/mL); ^c^ *p <* 0.05 compared with NLCTQ (5 μg/mL)

The formulation NLCTQ exhibited mutagenic effects in U87-MG cells, as evidenced by a significant increase of micronuclei, nuclear bridges, and nuclear buds at both tested concentrations (2.5 and 5 µg/mL) when compared with the negative control group (*p <* 0.05, Table 3). Temozolamide-treated cells at 50 and 100 µg/mL also showed differences in these markers relative to each other, whereas NLCQb did not induce alterations (*p >* 0.05). In addition, NLCTQ exhibited a cytotoxic effect, demonstrated by a reduction in the NDI and NDIC (Table 3, Fig. 8). Furthermore, an increase of apoptosis and necrosis was observed in NLCTQ-treated cells (2.5 µg/mL: 88.0 ± 1.7 and 21 ± 5.0; 5 µg/mL: 175.6 ± 3.7 and 89 ± 6.0, respectively) when compared to the NC (Fig. 9).

**Table 3:** Mutagenicity and cytotoxicity induced by free and nanoencapsulated TMZ in human glioblastoma cells (U87-MG) after 72 h of exposure, evaluated by the cytokinesis-block micronucleus (CBMN) assay

Treatment	Mutagenicity			Cytotoxicity	
	**Micronuclei**	**Bridges**	**Buds**	**NDI**	**NDIC**
Negative control	1.00 ± 1.00^bc^	1.00 ± 1.00^bc^	0.00 ± 0.00^bc^	3.21 ± 0.03^ab^	3.21 ± 0.03^abc^
TMZ 50 μg/mL	13.00 ± 1.73^a^	5.00 ± 1.00^ac^	7.00 ± 1.73^ac^	1.70 ± 0.01^a^	1.77 ± 0.02^ac^
TMZ 100 μg/mL	15.0 ± 5.19^a^	8.00 ± 1.00^ac^	7.00 ± 1.73^a^	1.22 ± 0.08^ac^	1.42 ± 0.06^ac^
NLCQb 2.5 μg/mL	0.66 ± 0.57^bc^	0.66 ± 1.15^bc^	0.00 ± 0.00^bc^	2.56 ± 0.01^abc^	2.56 ± 0.01^abc^
NLCQb 5 μg/mL	0.66 ± 0.57^bc^	0.33 ± 0.57^bc^	0.00 ± 0.00^bc^	2.54 ± 0.02^abc^	2.54 ± 0.02^abc^
NLCTQ 2.5 μg/mL	10.00 ± 1.00^ac^	9.00 ± 1.00^a^	11.67 ± 1.52^ab^	1.70 ± 0.02^ab^	1.81 ± 0.03^ab^
NLCTQ 5 μg/mL	19.33 ± 3.05^a^	12.33 ± 2.51^ab^	14.33 ± 2.08^ab^	1.78 ± 0.14^ab^	1.90 ± 0.01^ab^

Data are expressed as mean ± S.D. from three independent experiments (*n =* 3). ^a^ p < 0.05 compared with the negative control by one-way ANOVA followed by Tukey; ^b^ *p < *0.05 compared with TMZ (100 μg/mL); ^c^ *p <* 0.05 compared with NLCTQ (5 μg/mL). TMZ: temozolomide. NLCQb: empty chitosan-functionalized nanostructured lipid carriers. NLCTQ: chitosan-functionalized nanostructured lipid carriers loaded with temozolomide. IDN: nuclear division index. IDNC: nuclear division index considering cytotoxicity.

### TMZ causes cell death

**Fig. 10:**
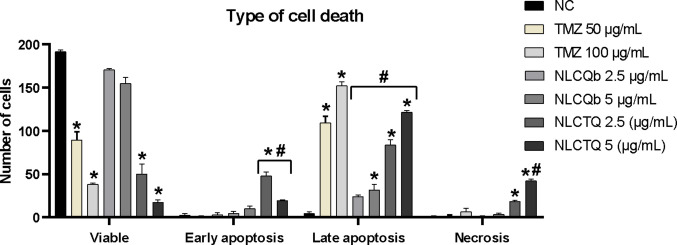
Cell death pattern assessed by fluorescence microscopy in U87-MG glioblastoma cells treated with free TMZ (50 and 100 μg/mL), empty chitosan-functionalized lipid nanocarriers (NLCQb: 2.5 and 5 μg/mL), or and TMZ encapsulated in chitosan-functionalized lipid nanocarriers (NLCTQ: 2.5 and 5 μg/mL). Data are expressed as mean ± S.D. from three independent experiments (*n = *3). Statistical analysis was performed using two-way ANOVA followed by Tukey. * indicates a significant difference (*p <* 0.05) compared with the negative control (NC); ^#^ indicates a significant difference (*p <* 0.05) compared with free TMZ 100 μg/mL

Analysis of U87-MG cells indicated that all treatments, with the exception of the empty nanocarriers (NLCQb: 2.5 µg/mL: 170.67 ± 1.16; 5 µg/mL: 154.67 ± 7.23), diminished the number of viable cells relative to the negative control (191.33 ± 2.08) (*p <* 0.05, Fig. 10). The reduction was most pronounced in groups treated with TMZ at 100 µg/mL (38.00 ± 1.73) and NLCTQ (2.5 µg/mL: 50.00 ± 11.53; 5 µg/mL: 17.33 ± 3.06). Furthermore, NLCTQ at concentrations of 2.5 µg/mL (48.00 ± 4.58) and 5 µg/mL (19.33 ± 1.16) elevated early apoptotic events relative to the negative control (NC) (2.67 ± 1.53) and TMZ at 100 µg/mL (3.00 ± 2.65). All treatments, with the exception of NLCQb at 2.5 µg/mL (24.00 ± 2.00), triggered late apoptosis compared to the negative control (NC) (4.67 ± 1.53, *p < *0.05).

**Fig. 11:**
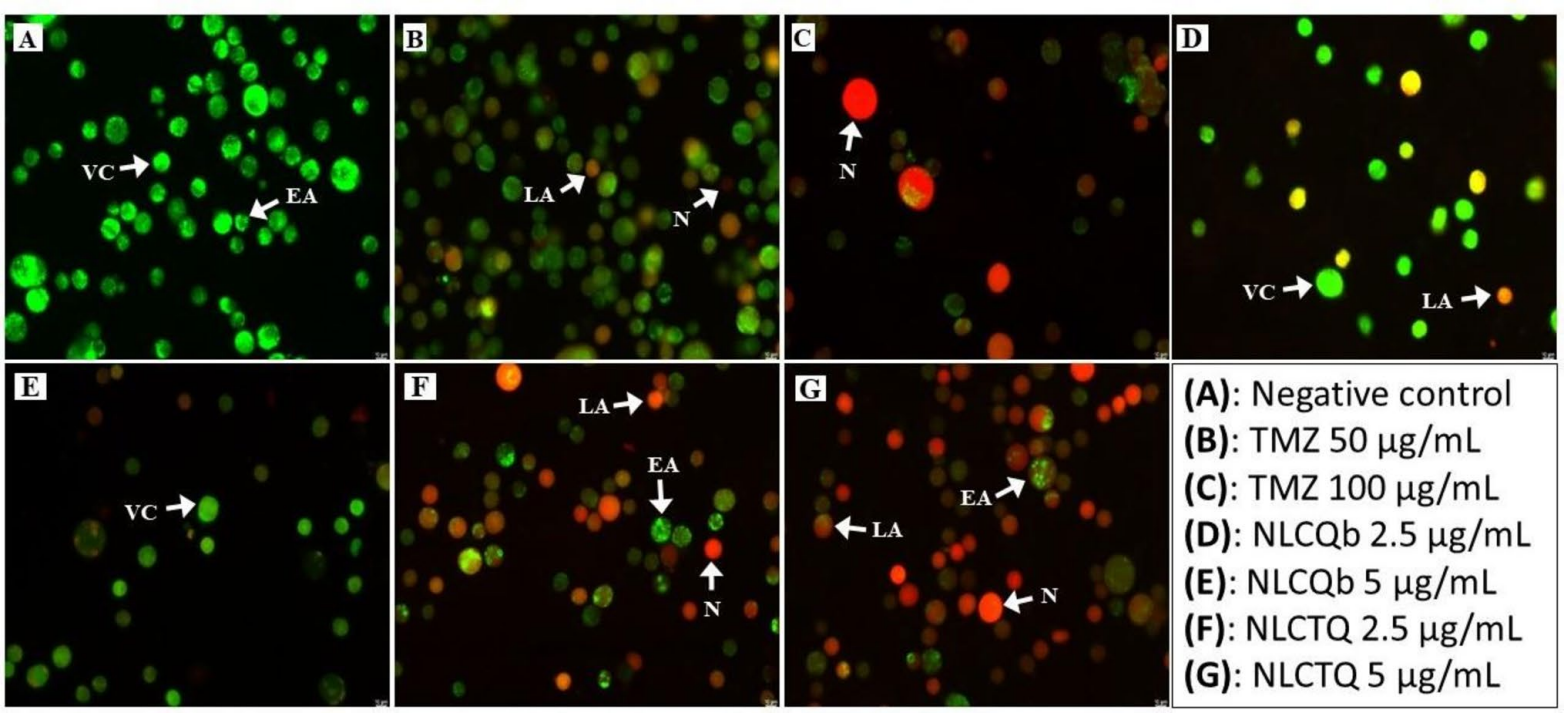
Profile of human U87-MG glioblastoma cells treated with free TMZ, NLCQb, and NLCTQ. Viable cells (VC); Early apoptosis (EA); Late apoptosis (LA); Necrosis (N)

The administration of NLCTQ at concentrations of 2.5 µg/mL (18.33 ± 1.53) and 5 µg/mL (42.00 ± 2.00) led to increased necrosis, with the 5 µg/mL concentration (42.00 ± 2.00) exhibiting a statistically significant difference (*p <* 0.05) compared to TMZ-treated cells at 100 µg/mL (6.67 ± 4.16), thereby indicating a more pronounced necrotic induction by this formulation at the elevated concentration (Fig. 10; Fig. 11).

## Discussion

As a target drug, TMZ presents important pharmacokinetic limitations that compromise its therapeutic efficacy. The drug has a short half-life (approximately 1.8—2 h), undergoes rapid degradation at physiological pH, and is quickly eliminated from the body. As a consequence, only a limited dose fraction reaches the brain, and less than 1% remains intact. Such reduced bioavailability makes the therapeutic effect highly dose-dependent. Therefore, it requires a lot of TMZ to have a therapeutic effect, which can cause hematological toxicity (Singh et al. 2025). In this context, the development of nanostructured delivery systems emerges as a promising strategy to overcome these limitations by improving drug stability, bioavailability, and brain delivery.

Herein, we demonstrate that nanoencapsulation of TMZ in chitosan-functionalized lipid carriers consistently enhanced antitumor efficacy of free TMZ. The formulation exhibited increased cytotoxicity, indicating that a nanostructured approach may offer relevant advantages over conventional chemotherapy. Particle size, a critical parameter for CNS delivery, remained below 155 nm for both NLCQb and NLCTQ, a suitable profile for crossing the BBB. Particles up to 200 nm exhibit improved systemic circulation and a greater ability to cross the BBB due to facilitated endocytosis and transcytosis in endothelial cells (El-Enin et al. 2022; Ghouri et al. 2022). The PdI values obtained indicated a homogeneous size distribution, a necessary condition for the stability and reproducibility of nanoformulations (Danaei et al. 2018). The study of Zardini et al. (2018) showed that the combination of Tween® 80 and lecithin favors reduced PdI, while values below 0.3 are ideal for controlled drug delivery systems, contributing to greater consistency in therapeutic performance (Colmenares et al. 2018).

The positive zeta potential of NLCQb and NLCTQ suggested adequate colloidal stability. NLCs typically exhibit a negative surface charge due to the lipid composition. Despite the presence of lecithin and hydroxyl groups from Tween® 80 (Meshram; Ranpise, 2024; Kim et al. 2023; Zardini et al. 2018), the positive charge observed here relies on protonated amine groups of CS. These groups increase stability and enhance electrostatic interactions with negatively charged cellular membranes, which may promote higher cellular uptake by glioblastoma cells (Tian et al. 2024; Oudih et al. 2023; Nguyen et al. 2017).

Despite adequate drug loading, the encapsulation efficiency was relatively low (39%), indicating that a larger fraction of the drug was not incorporated. Previous studies have reported higher encapsulation efficiencies for TMZ-loaded NLCs, generally associated with greater lipophilic solubility and optimization of formulation conditions (Zhang et al. 2018; Chen et al. 2016). The low efficiency observed in the present study may be related to the limited solubility of TMZ in the lipids employed, the drug-to-lipid ratio, or the use of CS, which may have affected drug partitioning. These factors highlight the need for formulation adjustments to improve TMZ incorporation (Zhang et al. 2018; Chen et al. 2016; Khan et al. 2016; Wu et al. 2016).

The encapsulation efficiency obtained for NLCTQ was 39%, a considerable value for hydrophobic matrix-based nanoformulations carrying hydrophilic drugs. Previous studies have reported that values ranging from 20 to 50% are commonly observed and considered adequate for this type of nanosystem (Marinelli et al. 2022; Özdal et al. 2022; Yalçin et al. 2021). However, some studies have reported higher encapsulation efficiencies for TMZ-loaded NLCs (Zhang et al. 2018; Chen et al. 2016). Importantly, when compared to recent TMZ nanocarriers, NLCTQ shows a more favorable physicochemical profile. Polymeric nanoparticles described by Şahin et al. (2025) exhibited particle sizes between 142.4 and 153.8 nm, whereas NLCTQ presented a smaller size (138.2 nm), which may favor cellular uptake and transport across the blood–brain barrier. These systems showed a zeta potential of −7.9 mV, a value associated with the flocculation threshold (Şahin et al. 2025). In contrast, NLCTQ exhibited higher positive zeta potential (+ 28.6 mV), suggesting improved colloidal stability and stronger electrostatic interaction with negatively charged cell membranes. A similar trend was observed when comparing NLCTQ with other chitosan-based nanostructures for TMZ delivery. Duarte et al. (2024) reported nanoemulsions with zeta potential values around + 20 mV, and NLCTQ revealed similar values (+ 28.6 mV), indicating good potential for membrane interaction.

The stability profile observed in the present study contrasts with the inherent instability commonly reported for nanostructured lipid carriers. Haider et al. (2020) describe NLCs as dynamic systems prone to aggregation, while Ortiz et al. (2021) reported particle size increases (~ 40 nm) at elevated temperatures. In contrast, no increase in particle size was observed at 4, 25, or 37 °C, with both formulations maintaining nanoscale dimensions and low PdI values over 60 days. Notably, at 37 °C, a reduction in particle size was observed, suggesting structural reorganization rather than instability. This behavior may be attributed to the use of Tween® 80 and lecithin, as well as to the lipid matrix composition, since less crystalline solid–liquid lipid mixtures form more disordered structures that improve drug accommodation and favor smaller, more stable particles over time (Da Silva et al. 2022). Furthermore, the increase in zeta potential at 37 °C indicates enhanced electrostatic repulsion between particles, contributing to colloidal stability and preventing aggregation, consistent with the low PdI values observed (Cheng et al. 2024).

In terms of cytotoxicity, IC₅₀ values indicated that NLCTQ outperformed free TMZ, confirming the ability of nanoencapsulation to enhance pharmacological effects, as evidenced by a significant difference of approximately 55-fold between NLCTQ (IC₅₀ of 4.79 µg/mL) and free TMZ (266 µg/mL). Additionally, Wang et al. (2021) reported anti-EphA3-functionalized gold nanoparticles loaded with TMZ with an IC₅₀ of 64.06 µM on TMZ-resistant glioma cells. In comparison, NLCTQ presented an IC₅₀ of 4.79 µg/mL (≈ 24.7 µM) on U87-MG cells. These findings indicate that NLCTQ achieves cytotoxic effects at substantially lower concentrations. Trypan blue exclusion assays corroborated the MTT findings, reinforcing the dose-dependent nature of the cytotoxic effect. As observed in nanocarrier systems with sustained drug release (Yadav et al. 2025; De Oliveira et al. 2024), these results suggest that NLCTQ may prolong the therapeutic effect and support further in vivo investigations.

To assess the antiproliferative effects of TMZ on in vitro 3D tumors, nanoencapsulated TMZ (NLCTQ) was administered to tumor spheroids, resulting in significant spheroid size reduction at a concentration of 5 μg/mL. In contrast, Marino et al. (2019) showed that TMZ in magnetic nanovectors did not induce cytotoxicity in U87-MG spheroids, even at elevated concentrations (167 μg/mL). Spheroids serve as an effective model for replicating tumor-like attributes, as they have molecular traits and structural organization comparable to solid tumors, featuring a necrotic center and a highly proliferative peripheral cell layer (Coelho et al. 2024).

The comet assay conducted with NLCTQ demonstrated an increase in DNA strand breaks at concentrations up to 20-fold lower than those needed to achieve similar effects with free TMZ. Zhu et al. (2023) reported TMZ-induced genotoxicity at elevated concentrations (200 µM = 38.8 µg/mL), whereas NLCTQ caused significant DNA damage at just 2.5 and 5 µg/mL. This difference in potency is likely due to several factors, including enhanced cellular uptake (Aibani et al. 2021), protection against hydrolytic degradation (Nazaruk et al. 2024), controlled drug release (Iturrioz-Rodríguez et al. 2023), and/or increased electrostatic affinity provided by CS (Aibani et al. 2021). Collectively, these elements contribute to the heightened genotoxic effects of NLCTQ.

The literature outlines specific mechanisms of drug absorption facilitated by chitosan-functionalized nanocarriers and their interactions with cells. Cellular uptake is attributed to the positive surface charge of chitosan (CS), which interacts with negatively charged cell membranes, thereby promoting cellular internalization. This process is partially linked to the “proton sponge” effect, a biophysical mechanism that enhances the uptake of positively charged nanoparticles (Megahed et al. 2022). Additionally, Fahmy et al. (2022) demonstrated that CS-based nanoparticles significantly increased cytotoxic effects, as indicated by the induction of apoptosis and necrosis.

Besides facilitating cellular internalization, CS coating improves interactions with biological barriers. Duarte et al. (2024) showed that CS-coated nanoformulations have mucoadhesive interactions with in vitro mucin. Mucin is a negatively charged glycoprotein overexpressed in glioma cells and associated with poor prognosis (Machado et al. 2025). CS interacts with mucin, alters the surface charge of the nanoformulation, and promotes anchoring effects on cellular surfaces. This interaction increases residence time and enhances absorption across biological membranes (Duarte et al. 2024). In addition, CS can interact with anionic molecules on cell surfaces and mucosal membranes, such as claudin proteins in tight junctions, modulating membrane permeability and facilitating nanoparticle internalization and intracellular transport (Ma et al. 2022; Yu et al. 2019). Together, the proton sponge effect, mucin interaction (anchoring mechanism), and modulation of cellular permeability promote controlled drug release and enhance TMZ absorption by U87-MG cells, which results in better intracellular availability, cytotoxicity, and alkylating activity.

TMZ is an alkylating agent that induces both single- and double-strand DNA breaks, as well as alkali-labile sites. These types of damage can be detected using the alkaline comet assay (Collins et al. 2023; Lu et al. 2017). Initially, this damage may lead to reversible injuries, as wild-type cells possess DNA repair mechanisms capable of restoring genomic integrity after exposure to the drug (Nandhakumar et al. 2011). Research by Castro et al. (2015) indicates that U87-MG cells exhibit prolonged survival even when exposed to 25 µM TMZ (equivalent to 4.9 µg/mL), suggesting a lower sensitivity to free TMZ. However, it is important to note that the comet assay does not directly measure cell viability or cell death; rather, it evaluates the extent of genotoxic damage, which is visualized through the formation of comet “tails” during electrophoresis (Collins et al. 2023; Pu et al. 2015). Therefore, while NLCTQ caused significant DNA damage, examining its effects on cell death patterns is essential.

Dual AO/PI staining showed that NLCTQ significantly reduced cell viability at concentrations of 2.5 and 5 µg/mL, demonstrating an efficacy comparable to that of free TMZ at 100 µg/mL. In contrast to free TMZ, only NLCTQ was capable of inducing early apoptosis at both concentrations. These findings indicate that NLCTQ surpasses the thresholds necessary for inducing cell death (Stratenwerth et al. 2021). Our results confirm the low sensitivity of U87-MG cells to free TMZ, as these cells have been observed to survive even after experiencing intermediate levels of DNA damage (Castro et al. 2015). In this investigation, free TMZ at concentrations of 100 µg/mL or less induced genotoxicity without a significant reduction in cell viability, suggesting the presence of repaired sublethal genotoxic damage (Chatterjee; Walker et al. 2017). Therefore, the extent of DNA alkylation caused by free TMZ is insufficient to elicit a lethal effect on the cells (Stratenwerth et al. 2021).

The low cytotoxic efficacy of TMZ at reduced concentrations can be related to its chemical instability. Following hydrolysis at physiological pH, the drug generates the intermediate MTIC, which decomposes into a methyldiazonium cation (responsible for DNA alkylation) and the inactive metabolite 5-aminoimidazole-4-carboxamide (AIC) (Campos-Sandoval et al. 2021). However, the active fraction is highly unstable, and its intracellular availability is limited. Moreover, the concentration of free TMZ that effectively reaches brain tissue is relatively low (1–10 µM), which probably impacts negatively on cell death (Stratenwerth et al. 2021).

At high concentrations (200 µg/mL), free TMZ significantly reduced cell viability (~ 66%), indicating that overcoming intrinsic U87-MG resistance needs doses that are clinically impractical due to systemic adverse effects. Furthermore, as resistance develops, the IC₅₀ of TMZ often surpasses 100 µM (19.4 µg/mL), which is not suitable for clinical use (Karve et al. 2024). These findings align with the previously described results, where treatment with NLCTQ at 5 µg/mL induced late apoptosis, while free TMZ exhibited comparable effects at concentrations 20 times higher. Collectively, these results suggest the presence of a concentration threshold (Stratenwerth et al. 2021), although at levels that are not clinically feasible. Moreover, the antitumor effect of TMZ involves the formation of methyl adducts in DNA bases, including O6-guanine (O6-MeG), N7-guanine (N7-MeG), and N3-adenine (N3-MeA), which lead to severe lesions and, if left unrepaired, contribute to genomic instability (Stepanenko et al. 2016; Fan et al. 2013).

NLCTQ also caused concentration-dependent reduction of NDI and NDI-C when compared with TMZ 100 µg/mL due to chromosomal instability, such as micronuclei, nucleoplasmic bridges, and nuclear buds. These events reflect the cytogenetic phenotype of breakage–fusion–bridge cycles, initiated by dicentric chromosomes and gene amplification (Fenech 2020). Thus, the presence of these biomarkers may be attributed to the clastogenic action of TMZ and double-strand breaks (Avlasevich et al. 2021). Despite rare studies with the CBMN assay of TMZ-treated gliomas, Pérès et al. (2014) also demonstrated TMZ promotes micronucleus formation, associated with instability that culminates predominantly in apoptosis and, to a lesser extent, necrosis in U87-MG cells.

Although the present findings highlight the therapeutic potential of NLCTQ, some limitations inherent should be addressed. In vitro experimental conditions do not fully reproduce the complexity of the in vivo tumor microenvironment, particularly vascularization, immune system interactions, and biological barriers. In vitro models do not adequately replicate the BBB, a critical obstacle that limits drug penetration into the brain and represents a major challenge for glioblastoma treatment (Iturrioz-Rodríguez et al. 2023; Janjua et al. 2023). Moreover, these models do not fully capture the intra- and intertumoral heterogeneity that underlies tumor progression, therapeutic resistance, and disease recurrence (Jubelin et al. 2022). Although 3D spheroids represent an advance over conventional monolayer cultures by partially mimicking tumor architecture, these models still lack the full physiological complexity of living systems, especially vascularization and cellular crosstalking (Jensen and Teng 2020; Jubelin et al. 2022).

In addition to these model-related limitations, aspects related to carrier-associated safety should also be considered. At 5 µg/mL, the empty nanocarrier (NLCQb) affected some evaluated parameters, which may be attributed to residual solvent traces resulting from insufficient filtration during its preparation, indicating the necessity for optimization of purification steps. For NLCQb, no alterations were observed at the lower concentration (2.5 µg/mL). Importantly, NLCQb neither induced chromosomal instability nor apoptosis/necrosis.

## Conclusion

This study highlights that TMZ incorporation into chitosan-functionalized nanostructured lipid carriers represents a relevant strategy to improve its cytotoxic performance against glioblastoma cells. The presence of CS enhances cellular interactions and promoted drug uptake and improved TMZ cytotoxic effects under lower concentrations. Antiproliferative and genotoxic effects observed for NLCTQ support its ability to improve therapeutic outcomes and overcome intrinsic resistance of U87-MG cells. In addition, NLCTQ induces DNA damage and formation of chromosomal instability biomarkers, including micronuclei, nucleoplasmic bridges, and nuclear buds, which is directly associated with cell death. Future investigations should focus on in vivo validation, analysis of blood–brain barrier permeability, safety profiling, and formulation optimization. These findings reinforce the value of NLCTQ as a promising strategy to improve the pharmacological performance of TMZ and justify further in vivo investigations and formulation optimization.

## Supplementary Information

Below is the link to the electronic supplementary material.

ESM 1 (DOCX 124 KB)

## Data Availability

The data that support the findings of this study are available from the corresponding author (JMCS) upon reasonable request.
