# Supplementary material for: Synthesis and characterization of chitosan-functionalized nanostructured lipid carriers with temozolomide: cytotoxic effects and chromosomal instability in human glioblastoma cells
Source: Naunyn Schmiedebergs Arch Pharmacol. 2026 Apr 27;399(10):15395–414. doi: 10.1007/s00210-026-05365-y (PMC13391459; doi:10.1007/s00210-026-05365-y)
Supplement: Supplementary file 1 — (DOCX 124 KB) [file 210_2026_5365_MOESM1_ESM.docx]

**Supplementary data**

**Physicochemical Characteristics**

**Fig. S1.** Particle size distribution of NLC formulations

(A) Particle size distribution by intensity of NLCTQ and (B) particle size distribution by intensity of NLCQb, obtained by dynamic light scattering (DLS) using a Zetasizer Lab instrument (Malvern Instruments). Measurements were performed at 25 ºC with a detection angle of 90º. NLCTQ presented a Z-average diameter of 138.2 ± 1.4 nm and a polydispersity index (PdI) of 0.24 ± 0.02, while NLCQb showed Z-average diameter of 153.4 ± 2.2 nm and a PdI of 0.23 ± 0.02. The distributions indicate monodisperse systems, suggesting adequate uniformity of the formulations.


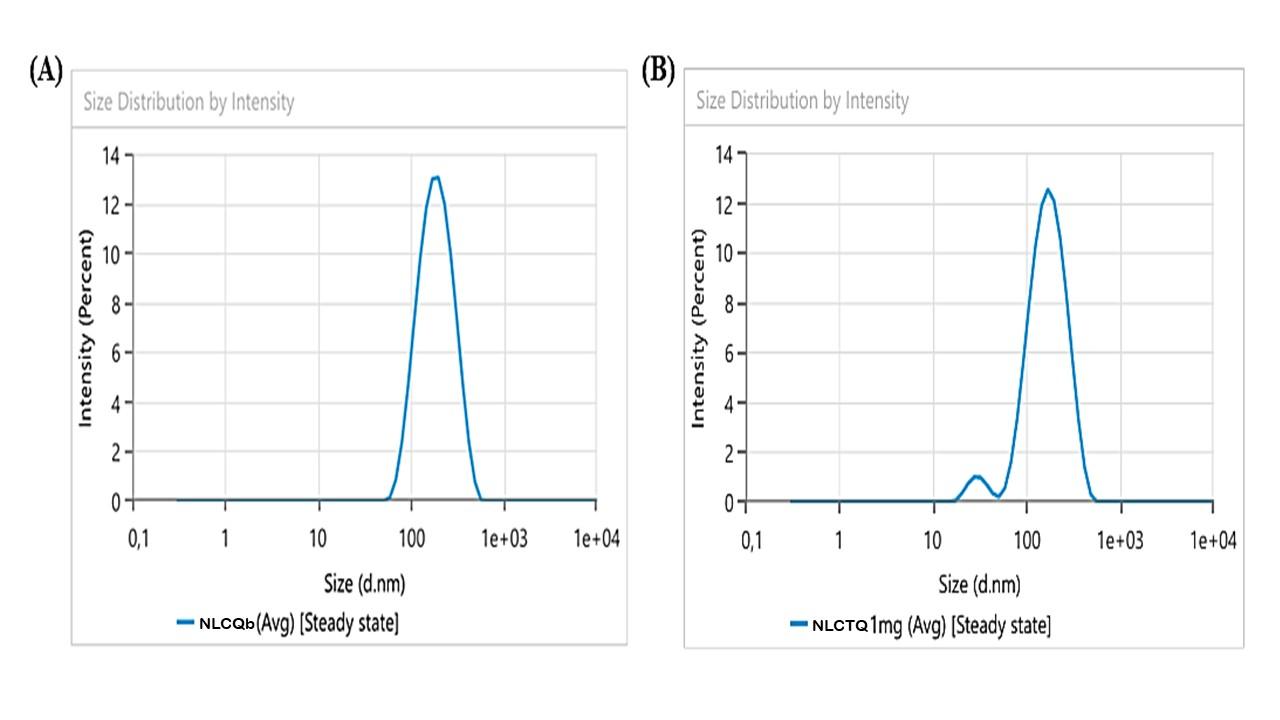


**Table S1.** Particle size and polydispersity index (PdI) of NLC formulations obtained by DLS

| Parameter | (A) NLCTQ | (B) NLCQb |
| --- | --- | --- |
| Z-average (nm) | 138.0 | 153.4 |
| PdI | 0.2432 | 0.2332 |
| Mean Count Rate (kcps) | 196.9 | 428.5 |
| Peak 1 (nm) | 184.9 | 200 |
| Peak 2 (nm) | 31.29 | - |
| Intercept | 0.9812 | 0.9711 |
| Fit Error | 0.002538 | 0.002328 |

Particle size distribution parameters were obtained by dynamic light scattering (DLS). Z-average corresponds to the intensity-weighted mean hydrodynamic diameter, while PdI indicates the width of the size distribution. Peak values represent distinct particle populations detected in the sample. Measurements were performed using a Zetasizer Lab instrument (Malvern Instruments) under standard conditions.

**Fig. S2.** Zeta potential distribution of NLC formulations

(A) Zeta potential distribution of NLCTQ and (B) Zeta potential distribution of NLCQb, determined by electrophoretic light scattering (ELS) using a Zetasizer Lab instrument (Malvern Instruments). Measurements were performed at 25 ºC. NLCTQ exhibited a mean Zeta potential of +28.6 ± 0.6 mV, while NLCQb showed a mean value of +26.2 ± 0.8 mV. The results indicate moderate colloidal stability based on the surface charge of the formulations.


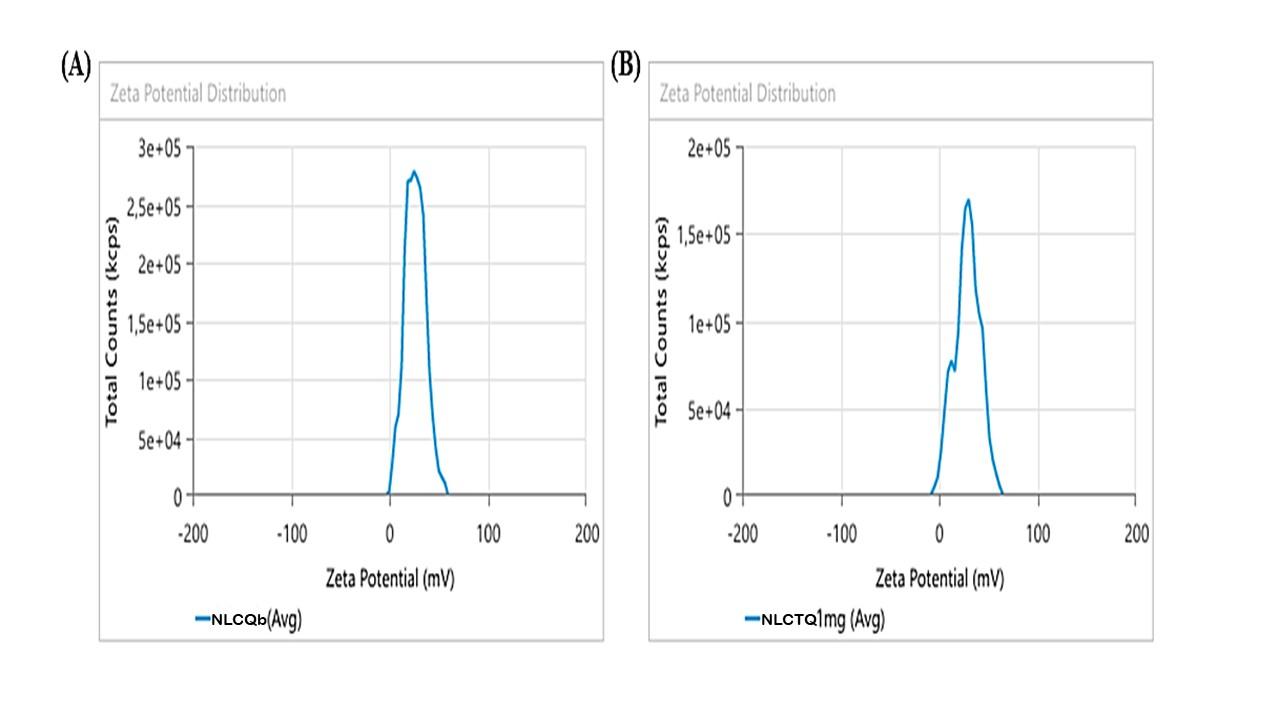


**Table S2.** Zeta potential parameters of NLC formulations

| **Parameter** | **(A) NLCTQ** | **(B) NLCQb** |
| --- | --- | --- |
| Zeta potential (mV) | 28.56 | 26.22 |
| Zeta potential (mV) | 12.99 | 10.35 |
| Conductivity (mS/cm) | 0.02561 | 0.0478 |
| Zeta Peak 1 Area (%) | 19.84 | 100 |
| Zeta Peak 1 (mV) | 9.769 | 26.22 |
| Zeta Peak 2 (mV) | 5.174 | 10.35 |
| Mean Count Rate (kcps) | 167.6 | 264.6 |

Zeta potential measurements were performed by electrophoretic light scattering. Zeta potential reflects the surface charge of the particles and is an indicator of colloidal stability. Values close to ± 30 mV suggest moderate to high stability. Measurements were performed under standard conditions using a Zetasizer Lab instrument (Malvern Instruments).
